# A Nomogram Based on a Multiparametric Ultrasound Radiomics Model for Discrimination Between Malignant and Benign Prostate Lesions

**DOI:** 10.3389/fonc.2021.610785

**Published:** 2021-03-02

**Authors:** Lei Liang, Xin Zhi, Ya Sun, Huarong Li, Jiajun Wang, Jingxu Xu, Jun Guo

**Affiliations:** ^1^ Department of Ultrasound, Aerospace Center Hospital, Beijing, China; ^2^ Department of Research Collaboration, R&D Center, Beijing Deepwise & League of PHD Technology Co., Ltd, Beijing, China

**Keywords:** radiomics, multiparametric ultrasound, clinical risk factors, machine learning, prostate cancer, nomogram model

## Abstract

**Objectives:**

To evaluate the potential of a clinical-based model, a multiparametric ultrasound-based radiomics model, and a clinical-radiomics combined model for predicting prostate cancer (PCa).

**Methods:**

A total of 112 patients with prostate lesions were included in this retrospective study. Among them, 58 patients had no prostate cancer detected by biopsy and 54 patients had prostate cancer. Clinical risk factors related to PCa (age, prostate volume, serum PSA, *etc*.) were collected in all patients. Prior to surgery, patients received transrectal ultrasound (TRUS), shear-wave elastography (SWE) and TRUS-guided prostate biopsy. We used the five-fold cross-validation method to verify the results of training and validation sets of different models. The images were manually delineated and registered. All modes of ultrasound radiomics were retrieved. Machine learning used the pathology of “12+X” biopsy as a reference to draw the benign and malignant regions of interest (ROI) through the application of LASSO regression. Three models were developed to predict the PCa: a clinical model, a multiparametric ultrasound-based radiomics model and a clinical-radiomics combined model. The diagnostic performance and clinical net benefit of each model were compared by receiver operating characteristic curve (ROC) analysis and decision curve.

**Results:**

The multiparametric ultrasound radiomics reached area under the curve (AUC) of 0.85 for predicting PCa, meanwhile, AUC of B-mode radiomics and SWE radiomics were 0.74 and 0.80, respectively. Additionally, the clinical-radiomics combined model (AUC: 0.90) achieved greater predictive efficacy than the radiomics model (AUC: 0.85) and clinical model (AUC: 0.84). The decision curve analysis also showed that the combined model had higher net benefits in a wide range of high risk threshold than either the radiomics model or the clinical model.

**Conclusions:**

Clinical-radiomics combined model can improve the accuracy of PCa predictions both in terms of diagnostic performance and clinical net benefit, compared with evaluating only clinical risk factors or radiomics score associated with PCa.

## Introduction

The incidence rate of prostate cancer (PCa) is rapidly increasing in China (1) and is the second most common cancer and the fifth leading cancer-related cause of death among males (2). As such, it has been one of the main health problems affecting many families. PCa screening has been studied in many randomized controlled trials, and different caveats have been proposed. Unfortunately, after detecting the serum level of prostate specific antigen (PSA) and/or performing a digital rectal examination, a 10- to 12-core systematic biopsy (3) is required by the standard diagnostic method. In addition to the complications related to this procedure (4), it has been reported that underestimation and overtreatment are high (5).Therefore, in order to avoid unnecessary trauma, the accuracy of non-invasive diagnostic methods before prostate biopsy must be improved.

In general, patients with PCa are divided into the low, medium, or high risk groups based on the level of prostate specific antigen (PSA), pathological assessment/Gleason score (GS), and clinical stage (*i.e.* T stage) (6). Although free prostate-specific antigen (fPSA), total prostate-specific antigen (tPSA), and the ratio of free PSA to total PSA (f/tPSA) are frequently applied to clinical PCa detection and grading indicators, (7–9), which indicators are more appropriate for the diagnosis and classification of PCa remains a controversy, and no agreement has been reached (10, 11). Based on the European Urology Association treatment guidelines for PCa in 2017, it is recommended that patients suffering from GS <7 PCa undergo active surveillance and wait for observation. On the contrary, because there is an increased risk of exacerbation and shorter rate of survival among patients with GS ≥7 PCa, it is necessary to take timely measures (3). Therefore, accurate risk assessment is important to select the best treatment option for these patients.

Multi-parameter magnetic resonance imaging (mpMRI) has become an important tool for PCa risk assessment. In the European Urological Association’s 2019 guidelines, the application of pre-biopsy mpMRI is recommended in their diagnostic approach. Nevertheless, in addition to several intrinsic limitations of MRI, such as high cost, limited availability, and unrealistic clinical application, the learning curve of prostate imaging report and data system (PI-RADS), is steep and there is a high risk of inconsistency between operators (12).

Ultrasound is another cost-efficient, widely available, and practical potential candidate for PCa imaging. Although some ultrasound modalities, such as shear-wave elastography (SWE), have shown encouraging results, targeted biopsies using B-mode ultrasound remain inferior to systematic biopsies (13). A multiparametric method has the principle of imaging well-known multifocal and heterogeneous diseases such as PCa (14) which is applicable to MRI and ultrasound by extracting information from tissue texture, elasticity, or perfusion and other complementary biomarkers. However, until now, a multiparametric ultrasound method has rarely been studied (15). Furthermore, there is growing interest in the use of quantitative features called radiomics. According to the definition, radiomics acts as the high-throughput extraction of many medical imaging characteristics and their conversion into mined, high-dimensional data whose quantitative analysis offers unprecedented opportunities to improve clinical decision-making (16, 17).

In previous studies, the analysis of radiomics features focused on evaluating and classifying PCa lesions (18, 19) using mpMRI. However, transrectal B-mode ultrasound is also a common imaging method to examine the prostate. Additionally, it is considered that tissue stiffness acts as an important indicator of malignant tumor for SWE, and recent studies have shown that it can also be used to detect PCa (20). In this study, our main purpose is to verify the feasibility of multiparametric ultrasound radiomics in discriminating between malignant and benign prostate lesions. Nevertheless, no studies exist that combine the features of ultrasound radiomics with clinical factors for risk assessment.

In consequence, we constructed models according to the principle of multiparametric ultrasound radiomics in combination with clinical factors to predict PCa, and compared whether the combination of these methods helps to improve diagnostic efficiency.

## Methods

### Patients Enrolled in This Study

**Figure 1:**
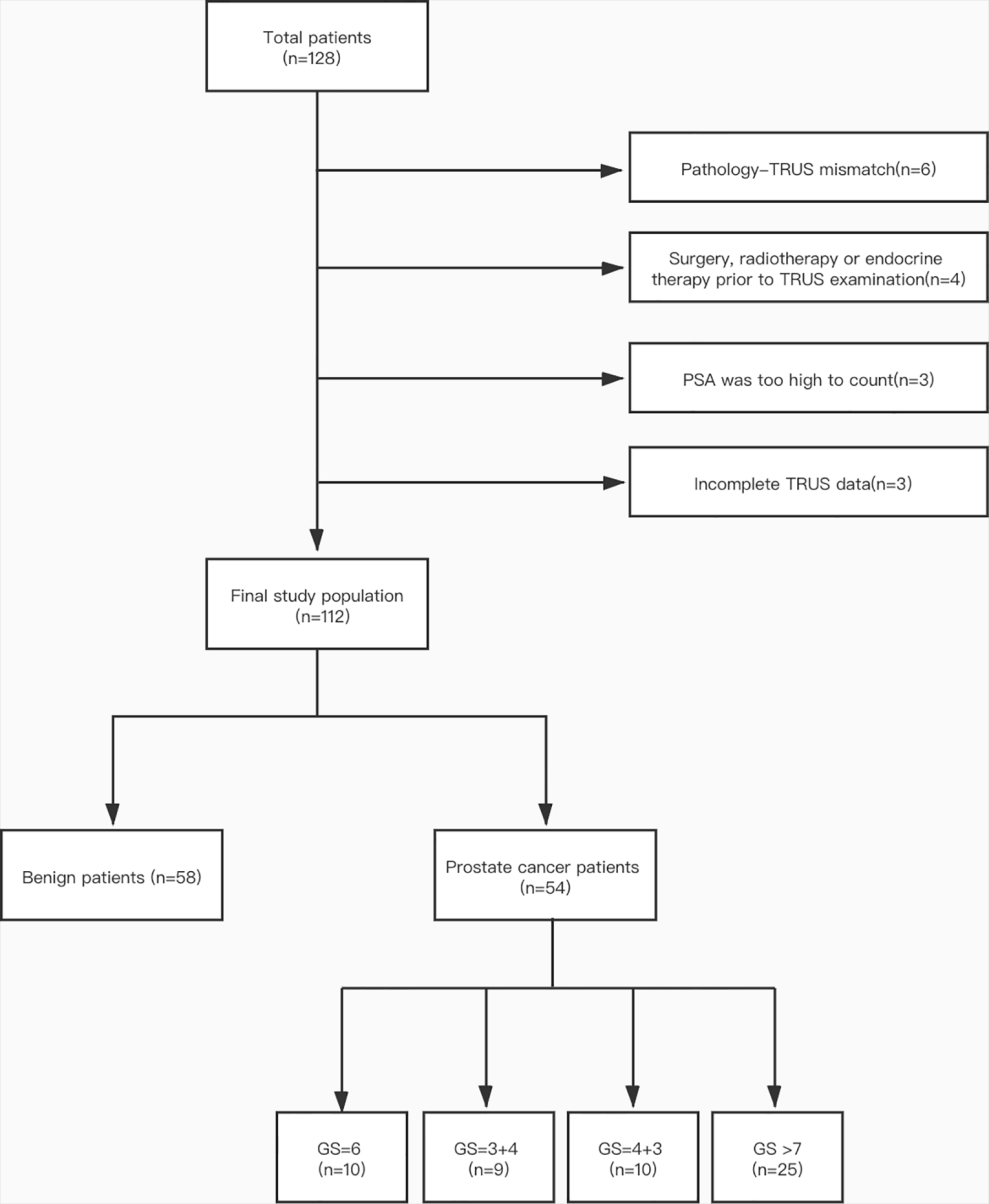
Patient selection flow chart.

The institutional Ethics Committee of our hospital approved this retrospective research, and an informed consent was been signed by all participants. A total of 128 patients were included in our hospital from July 2019 to November 2020. Inclusion criteria were as follows: (1) patients have clinical symptoms (frequency and urgency of urination, urination or dysuria pains) or enhanced PSA level; (2) patients have completed transrectal B-mode ultrasound and SWE examinations before receiving ultrasound-guided biopsy; (3) pathological results were confirmed through biopsy, and (4) patients with initial biopsy. Exclusion criteria were shown below: (1) it is difficult to describe pathological biopsy by transrectal ultrasound (TRUS) (according to pathological results, TRUS images fail to show lesion location) (n = 6); (2) surgery, radiotherapy or endocrine therapy prior to TRUS examination (n = 4); (3) PSA was too high to calculate (n = 3), or (4) incomplete TRUS data (lack of SWE data) (n = 3). Ultimately, the study population consisted of 112 patients including 58 PCa patients and 54 patients who do not show any histological evidence of cancer. **Figure 1** shows the details of patient selection.

### Clinical Data

Age, prostate volume (PV), serum PSA (including tPSA and fPSA), f/t PSA, Prostate-specific antigen density (PSAD), DRE result (normal *vs* abnormal), prostate biopsy pathology and other clinical information were collected from the patients selected. On the images of TRUS, PV was calculated as anteroposterior diameter × vertical diameter × transverse diameter× 0.52. PSAD was calculated as total PSA/PV.

### Ultrasound Image Data Acquisition

Each patient underwent B-mode ultrasound and SWE recording of the apical, middle, and bottom of the prostate. The examination was performed using an Aixplorer^®^ Ultrasound scanner (SuperSonic Imagine, Aix en Provence, France) equipped with a SE 12-3 transrectal probe.

After standard PV measurement and assessment of the prostate capsule and seminal vesicles, B-mode ultrasound was applied to slowly capture the transverse and sagittal scans of the whole prostate. Abnormal echo patterns (calcifications, cysts, and hypoechoic lesions) were recorded, and the pictures of the apical, middle, and bottom transverse plane of interest were determined and stored by the operator visually based on the anatomical shape of the prostate. If the prostate areas were considered to more suspicious than the anatomically selected imaging plane, these areas would be brought and stored to the field of view.

**Figure 2:**
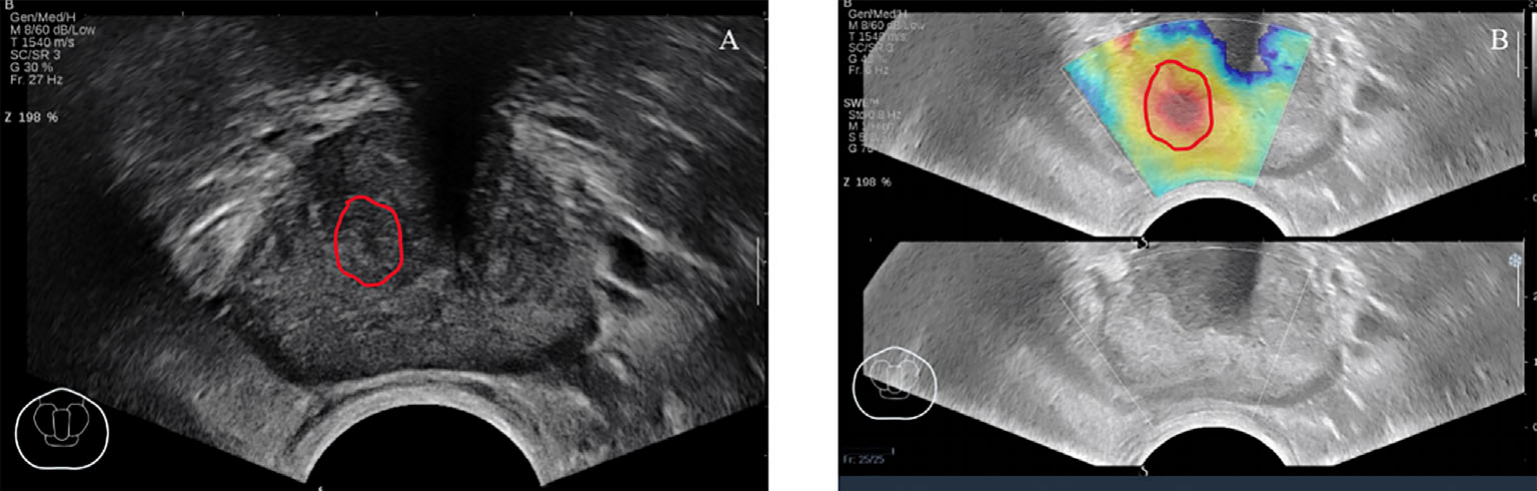
A 67-year-old patient had no obvious abnormal lesions in B-mode ultrasound **(A)**, SWE **(B)** showed that the local tissue became stiff, and the biopsy result was Gleason = 3 + 4. ROI was delineated under the guidance of the abnormal area of SWE.

If necessary, the settings specific to SWE (maximum penetration and suitable elasticity level) were reviewed and optimized before SWE imaging. The SWE box would be used to scan each pre-defined transverse plane in one side (left/right only) and both sides (whole plane; maximum prostate plane coverage). During each scanning, a stable signal is ensured in case of 5-s stay of the sensor remained in a stable position. After storing the pictures and cine loops, elastic values could be determined later. If prostate areas on the SWE outside the predetermined imaging plane were considered more suspicious, then these areas would also be taken into account. **Figure 2** shows an example of SWE.

We conducted a retrospective review of the image data and selected ultrasound image data in digital imaging and communications in medicine (DICOM) format to clearly show the maximum cross section of each lesion. The above image information and format were retained for later image segmentation.

### Biopsy Procedure and Pathology

All patients ceased taking anti-coagulants for one week before biopsy and took antibiotics for three days after biopsy. No local anesthetic was applied during the biopsy. The biopsies were performed by two sonographers with more than five years of biopsy experience. An Aixplorer^®^ Ultrasound scanner (SuperSonic Imagine, Aix en Provence, France) equipped with a SE 12-3 transrectal probe (end-fire) was applied. An 18-G biopsy gun with the length of 18 mm and a penetration depth of 22 mm was applied to perform the procedure (Bard Biopsy Systems, Tempe, Arizona, USA).

All patients underwent the “12+X” biopsy, which is a targeted biopsy for suspicious areas (combined with B-mode and SWE) on the basis of 12-core transrectal systematic biopsy. Systematic biopsy means that, according to the plan, the needle is inserted into 12 regions of the prostate (medial and lateral apex, medial and lateral mid prostate, and medial and lateral base in both lobes), with one needle in each region (21). In addition to the above-mentioned 12 needles, one to two needles were punctured in the suspicious area.

### Prostate Segmentation

**Figure 3:**
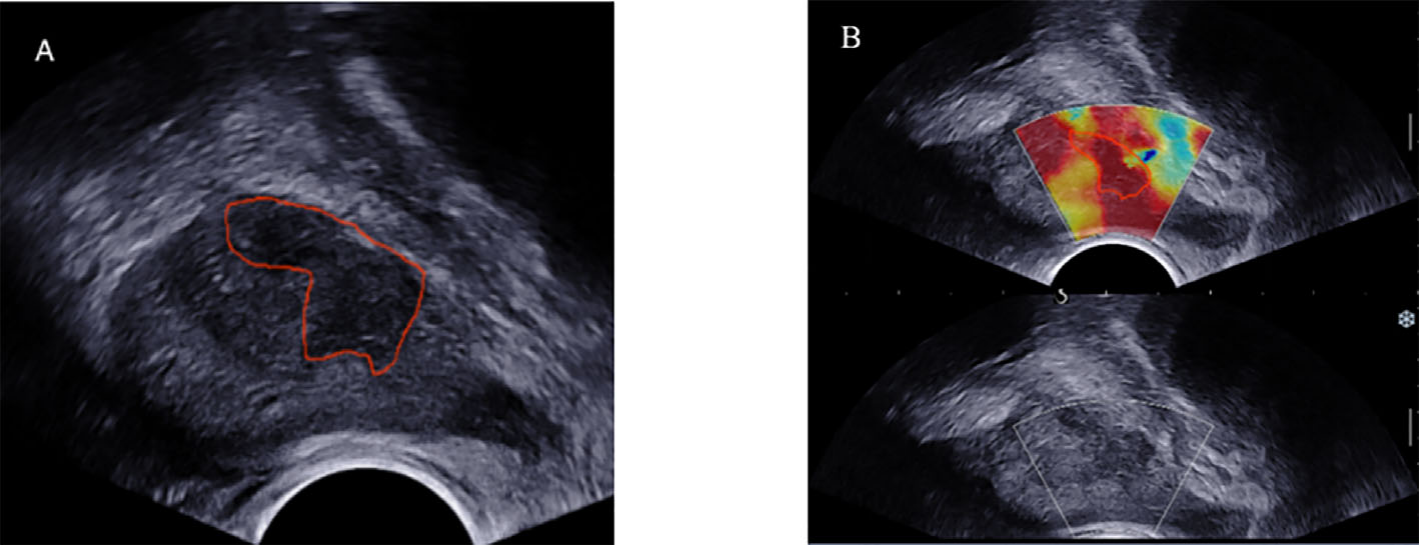
TRUS B-mode ultrasound imaging **(A)** and SWE imaging **(B)** from the same position of 74-year-old PCa patient (fPSA, 1.28 ng/ml; tPSA, 7.855 ng/ml; biopsy GS,4 + 3 = 7). ROI (red solid line) was outlined in the B-mode ultrasound and SWE.

We imported the images into the ITK-SNAP software (version 3.8.0) to manually draw the tumor boundary and determine the tumor region of interest (ROI). To ensure the consistency of the ROIs in the B-mode ultrasound and SWE images, the same criteria were applied to rigorously depict all the ROIs, and the same expert visually verified them. The following content shows the location and size of the lesion: (1) detailed records of prostate biopsy (puncture site and depth) and pathological findings were used to determine the location and nature of the lesion; (2) the description of pathology location matches the related lesion on the TRUS image, and (3) due to the uncertainty of tumor boundary in SWE images, ROIs of B-mode ultrasound images were applied to the corresponding SWE images. There is a notable aspect of ROI drawing: for multifocal PCa, biopsy pathology was applied to select and confirm the ROI of the lesion with the highest GS value; in the case of the same GSs, the ROI of the lesion with the largest diameter was used. **Figure 3** shows an example of lesion segmentation for enrolled patients. At the same time, special personnel were responsible for checking the accuracy of the segmentation and relevant pathological results.

The repeatability of feature extraction was assessed on the basis of intra-observer and inter-observer repeatability of lesion segmentation. In order to assess the repeatability of characteristic extraction between intra-observer and inter-observer, 40 patients were randomly selected and the ROI was delineated by two radiologists. Both radiologists had more than three years of experience in prostate ultrasound diagnosis.

### Radiomics Feature Extraction

This study used the Dr. Wise Multimodal Research Platform (https://keyan.deepwise.com) (Beijing Deepwise & League of PHD Technology Co., Ltd, 193 Beijing, China) for feature extraction. 1,218 features were extracted from ROI of B-mode and SWE; the extracted features were divided into seven categories: First Order Features, Shape Based, Gray-scale Co-occurrence Matrix(GLCM) Features, Gray-level Size Zone Matrix(GLSZM) Features, Gray-level Run Length Matrix (GLRLM) Features, Gray-Level Distance-Zone Matrix (GLDM) and Neighboring Gray Level Dependence matrix.

### Model Construction

Prostate lesions were identified by clinical elements and radiomics characteristics. We used the five-fold cross-validation method to verify the results of training sets and validation sets of different models. With regard to clinical elements, logistic regression models were established by univariate and multivariate logistic analyses to identify the relationship between clinical factors and prostate lesions. In terms of the multiparametric ultrasound-based radiomics model, we attempted to use six kinds of feature-screening methods including F-Test, Pearson Correlation Coefficient, Mutual Information, L1-based, Tree-based models, and Recursive Feature Elimination. Only one of the above methods was used for feature screening each time to build the model. Finally, the L1-based model was selected because of its optimal diagnostic performance. Through analyzing the logistic regression of the selected characteristics weighted by their coefficients, a formula called RAD-SCORE was generated. An integrated clinical-radiomics combined model with the weight of radiomics characteristics and clinical risk factors was established by using multivariate logistic regression and was presented in the form of nomogram.

### Statistical Analysis

When establishing the clinical model, the clinical factors were selected by applying univariate logistic regression, and the clinical model was established by introducing the clinical factors with *p <*0.05 into multivariate logistic regression. In logistic regression, the forward stepwise selection method was adopted. Finally, the clinical model was set up. The area under curve (AUC) with 95% confidence intervals (95% CIs) was used to quantify the performance of each model. Whether the AUC values of the three models were significantly different was determined by employing the DeLong test. The nomogram of the clinical-radiomics model was constructed to improve decision making. Decision curve analysis was conducted to determine the clinical usefulness of the clinical, radiomics and clinical-radiomics combined model. R software (version 4.0.2) and SPSS (version 23.0) were employed to perform analysis.

**Figure 4:**
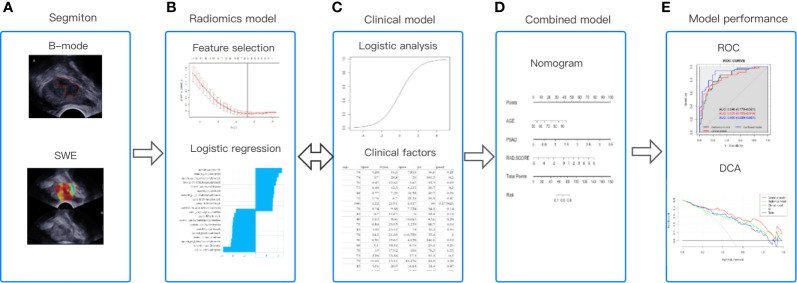
The workflow of this study. **(A)** Regions of interest were segmented from B-mode ultrasound and SWE. **(B)** The quantitative imaging texture features were extracted and selected to construct the radiomics model. **(C)** Clinical risk factors were used to establish the clinical model. **(D)** At the same time, the radiomics and clinical factors were added to construct the combined clinical-radiomics model. **(E)** ROC curve analysis and DCA were used to evaluate the performance of the model.

The entire workflow of this analysis was presented in **Figure 4**.

## Results

### Patient Characteristics

Among the 112 patients, 58 (51.7%) presented with benign lesions, and 54 (48.2%) were diagnosed with PCa. The GS results of all patients were as follows: 3 + 3 = 6 (10 cases); 3 + 4 = 7(nine cases); 4 + 3 = 7 (10 cases); >4 + 3 (25 cases). **Table 1** shows the features of all patients.

**Table 1:** Characteristics of all patients (n = 112).

Characteristics	Cohort of benign patients	Cohort of PCa patients
Number of patients, n	58	54
Age, years (median; IQR)	72.00(66.00–75.00)	78.00 (71.75–83.00)
PV, ml (median; IQR)	52.70(40.70–83.12)	43.70(34.08–58.83)
tPSA, ng/ml(median; IQR)	6.40 (4.62–10.58)	12.38 (8.85–62.00)
fPSA, ng/ml(median; IQR)	1.19(0.72–1.70)	1.92(1.13–7.63)
f/tPSA, % (median; IQR)	18.31(12.62–25.16)	16.52(11.20–20.85)
PSAD, ng/ml/ml (median; IQR)	0.12(0.07–0.20)	0.32(0.17–1.25)
Abnormal DRE, n (%)	32(55.2)	30(55.5)

### Clinical Model

With regard to clinical factors, tPSA, fPSA, and PSAD were important factors for the prediction of PCa based on the univariate logistic regression analysis. According to multivariate logistic analysis, age and PSAD were important (*p* < 0.05) as independent predictors. The outcomes of the univariate and multivariate logistic regression analysis were presented in **Table 2**. At the end, a logistic regression classifier was set up according to the clinical characteristics selected. The AUC of the training set was 0.88 (95% CI: 0.82–0.95), accuracy rate was 0.81, sensitivity was 0.75, and specificity was 0.87. The AUC of the validation set was 0.84 (95% CI: 0.76–0.91), accuracy rate was 0.76, sensitivity was 0.67 and specificity was 0.85.

**Table 2:** Univariate and multivariate logistic analysis results of clinical factors.

Clinical factors	Univariate logistic analysis results		Multivariate logistic analysis results	
	OR (95% CI)	*p* value	OR (95% CI)	*p* value
Age	1.071(1.019–1.126)	0.007	1.123(1.049–1.202)	0.001
fPSA	1.561(1.108–2.200)	0.011		0.907
tPSA	1.129(1.040–1.226)	0.004		0.563
f/t PSA	0.978(0.935–1.023)	0.339		
PV	0.994(0.983–1.006)	0.321		
PSAD	227.470 (7.374–7017.363)	0.002	550.945 (13.503–22479.425)	0.001
DRE results	1.141(0.348–3.734)	0.828		

### Radiomics Model

Intra-observer and inter-observer consistency for characteristic extraction were assessed by using intra-class and inter-class correlation coefficients (ICCs). Feature extraction of intra-observer and inter-observer showed good reproducibility, with intra-observer ICCs ranging from 0.78 to 0.85 and the inter-observer ICCs ranging from 0.75 to 0.88.

In the training set, after the LASSO algorithm was applied, 1,218 B-mode features were reduced to 20 risk predictors, and corresponding steps were also completed for the SWE data set. Then, after the lasso regression, the total 2,436 features of the two modes were reduced to 20 related features, and the Multiparametric RAD-SCORE was obtained. The sum of the weighted features of B-mode RAD-SCORE, SWE RAD-SCORE and Multiparametric RAD-SCORE are shown in **Appendix 1**.

**Figure 5:**
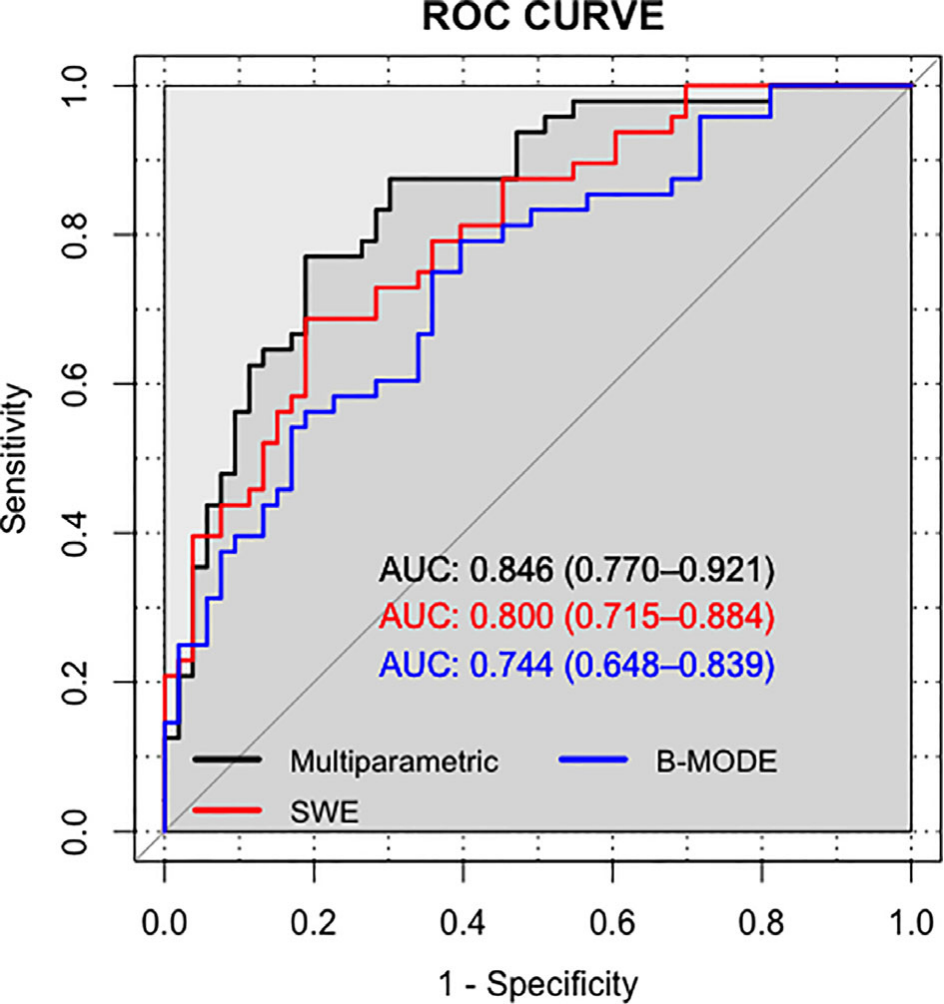
ROC of B-mode ultrasound, SWE, and Multiparametric.

The AUC values of the training set of the B-mode RAD-SCORE, SWE RAD-SCORE, and multiparametric RAD-SCORE were 0.97 (95% CI: 0.94–1.00), 0.97(95% CI: 0.94–1.00), and 1.00(95% CI: 0.949–1.00)), respectively. And the AUC values of the validation set were 0.74 (95% CI: 0.65–0.84), 0.80(95% CI: 0.72–0.88) and 0.85 (95% CI: 0.77–0.92), respectively. The ROC curves of the three models were shown in **Figure 5**.

### Clinical-Radiomics Combined Model

**Figure 6:**
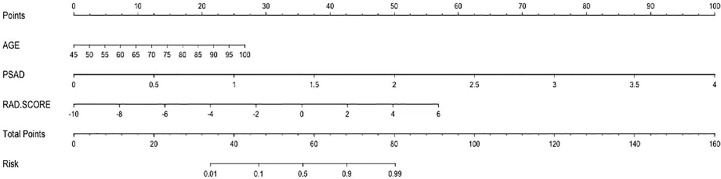
Nomogram of the combined model for predicting PCa.

**Figure 7:**
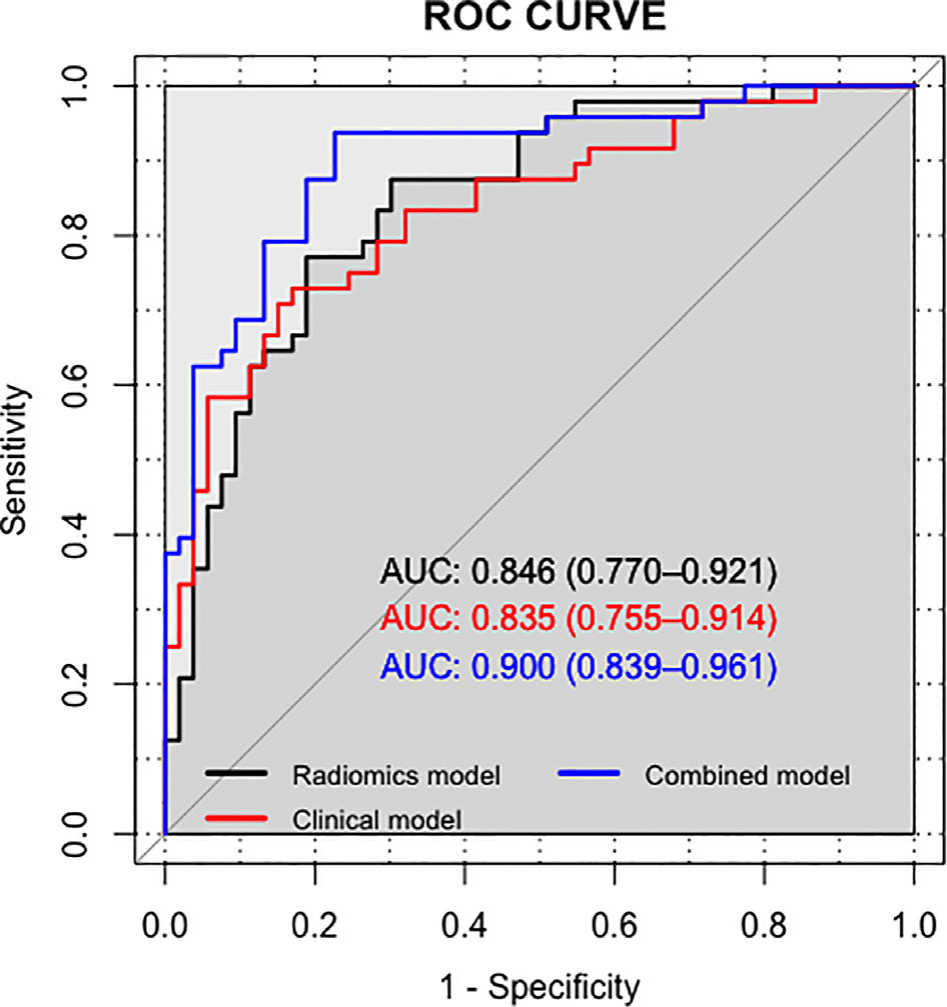
Comparison of ROC curves for differentiation of the three models for predicting PCa.

The nomogram of the clinical-radiomics combined model including age, PSAD, and RAD-SCORE is shown in **Figure 6**. The clinical-radiomics combined model displayed excellent predictive capacity with AUC of 0.91(95% CI: 0.86–0.97) in the training group and 0.89 (95% CI: 0.82–0.96) in the validation group. Despite showing the same diagnostic efficacy as the radiomics model, the clinical-radiomics combined model was better than the clinical model in diagnostic efficacy (*p* < 0.05). The AUC, accuracy, sensitivity, and specificity of the three models are compared and presented in **Table 3**. The ROC curves of the three models are compared in **Figure 7**.

**Table 3:** AUC results of the clinical, radiomics, and clinical-radiomics combined models for predicting PCa.

	Clinical model		Radiomics model		Clinical-radiomics combined model	
	Training set	Validation set	Training set	Validation set	Training set	Validation set
AUC (95% CI)	0.88 (0.82–0.95)	0.84 (0.76–0.91)	1.00 (0.99–1.00)	0.85 (0.77–0.92)	0.92 (0.86–0.97)	0.90 (0.84–0.96)
Accuracy	0.81	0.76	0.99	0.78	0.84	0.81
Sensitivity	0.75	0.67	1.00	0.77	0.79	0.75
Specificity	0.87	0.85	0.98	0.80	0.89	0.87

### Decision Curve

**Figure 8:**
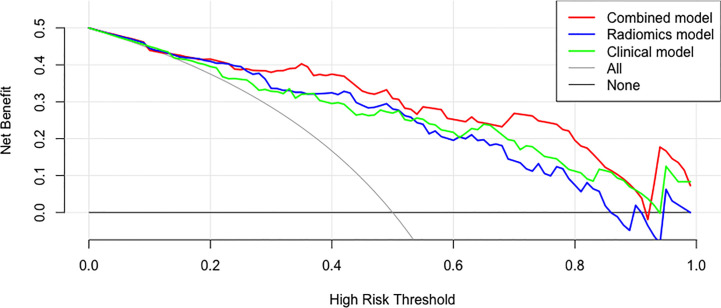
Decision curves of the clinical, radiomics, and clinical-radiomics combined models for predicting PCa.

The decision curves of the clinical model, the radiomics model, and the clinical-radiomics combined model are shown in **Figure 8**. According to the decision curve, the clinical-radiomics combined model was more beneficial in a wide range of high risk threshold than the clinical and radiomics models alone in predicting PCa.

## Discussion

In this study, the development of a multiparametric radiomics classifier was reported for the classification of prostate lesions on basis of co-registration of B-mode ultrasound and SWE. The radiomics model was established by extracting quantitative imaging characteristics and effectively choosing these characteristics. Subsequently, a clinical-radiomics combined model was developed by combining the RAD-SCORE with clinical factors. According to the results, clinical-radiomics combined model can improve the accuracy of PCa predictions both in terms of diagnostic performance and clinical net benefit, compared with evaluating only clinical risk factors or radiomics score associated with PCa.

### Clinical Factors Associated With PCa

In previous studies, prostate related clinical factors were identified by researchers to assist with diagnosing PCa and assessing its invasiveness. However, agreement has not been reached. Niu et al. showed that age, PI-RADS version 2 score, and adjusted PSAD were independent predictors of high-grade PCa (HGPCa), with an AUC of 0.83 (22). Fang et al. predicted the presence of PCa and HGPCa by applying clinical factors (age, PSA, fPSA, PV, and TRUS) with or without MRI outcomes. The AUC values for the prediction of PCa with or without MRI were 0.875 and 0.841, respectively, while those for the prediction of HGPCa were 0.872 and 0.850, respectively (23). In a study by Li et al., patients with benign lesions and GS = 6 were grouped into clinically insignificant PCa. After univariate and multivariate logistic analysis, the results showed that age, tPSA, fPSA and clinical factors were important factors for predicting significantly important PCa, with an AUC value of 0.842 (24). Our study also showed that age and PSAD were important predictors of PCa. Considering the total level of PSA and prostate volume, PSAD may have more individualized significance than serum PSA level, as previously reported in the literature (25). However, our results were not completely consistent with the previous studies mentioned above. This may be due to the different clinical factors selected in each study and inconsistent case grouping. Therefore, a more simple and accurate method should be developed for the grouping and scoring of prostate cancer patients.

### Some Radiomics Features That Can Discriminate Prostate Lesions

According to the previous studies, hypoechoic lesions have a relatively low ability to predict PCa (26, 27). This may be due to the subjective choice of hypoechoic lesions by the operator, thereby making its reproducibility and representativeness problematic (26, 28). Although hypoechoic lesions of B-mode ultrasound cannot be used as a predictor of PCa, some studies have shown that PCa with hypoechoic lesions may represent high-grade Gleason. According to the report by Nakano Junqueira et al., patients who have received prostatectomy with hypoechoic lesions experienced greatly worse prognosis than those without hypoechoic lesions, despite great differences in Gleason score, PSA, and percentage of positive cores (29). However, previous studies have limitations in the repeatability and representativeness of the quantitative expression of outcomes. The significance of our study is that radiomics can provide a numerical value by quantitative analysis of gray level. Our results showed that the Mean@b (*i.e.* The average gray level intensity within the ROI) of PCa was lower than that of benign lesions, and the Mean@b can be used as a predictor of PCa. Moreover, the average gray level of different nodules can be obtained by using radiomics, which can provide an objective index for the prediction of PCa.

Variations in cellular composition, fluid content, collagen level, and fibromuscular stroma in different types of prostate lesions may be reflected by quantitative analysis through radiomics. Our study showed that patients with benign lesions displayed lower values in Correlation, which is a value between 0 (uncorrelated) and 1 (perfectly correlated) indicating the linear dependence of gray level values on their respective voxels, with high relative weight. In another parameter of homogeneity, benign nodules displayed higher levels of Gray Level Non-Uniformity Normalized (GLNN), that is the changes of gray-level intensity values in the image, with a lower value reflecting a larger similarity in intensity values, than that of PCa. The above results indicated that the consistency in benign prostatic lesions was lower than that in PCa. The most common benign nodular lesion in the prostate is benign prostatic hyperplasia (BPH), which is usually accompanied by an inflammatory reaction. Inflammatory cells and pro-inflammatory cytokines can be detected in the histopathology of the resected BPH specimens (30). In addition, studies have confirmed that BPH is often characterized by the increased number of cells of different components, not only in the number of gland cells, but also in the number of smooth muscle cells, or may even consist entirely of stromal nodules (31). However, the pathological changes of PCa are mostly because of the increase of the number of cancer cells and the changes of extracellular space (24). Therefore, we speculated that the heterogeneity of cellular components may be the reason for the lower consistency of ultrasound radiomics in benign prostatic lesions than in PCa.

Even before morphological changes are detected by MRI and ultrasound examination, the stiffness of prostate tissue changes early with the effect of desmoplastic reaction or cancer cells infiltrating into the interstitial tissues, leading to PCa tissue feeling harder than normal tissue (32–34). Therefore, current guidelines suggest that SWE can be used as an auxiliary means for TRUS to detect PCa (35). Considering the above factors, we incorporated SWE into the radiomics model in this study. As a rule, in SWE images, the redder the color, the stiffer the tissue, and in the radiomics standards, the redder the color, the higher the gray level. Our study showed that the value of Mean@SWE in PCa was higher, indicating that the nodules of PCa are stiffer, which is consistent with the results of previous studies. Our results also showed that the multiparametric radiomics model combined with B-mode ultrasound and SWE had better diagnostic performance than the single-parametric model, and the AUC can reach 0.85.

### Some Advantages of Our Study: Widely Used Radiomics Features and Nomograms

Recently, some studies have compared or combined radiomics, including mpMRI, CT, and prostate specific membrane antigen-positron emission computed tomography (PSMA-PET), with common approaches to assess the diagnostic value of prostate lesions (25, 36, 37). The research focused on the use of mpMRI combined with PI-RADS, which can accurately characterize the prostate index lesions derived from mpMRI by using quantitative image data (38). Another important examination is ultrasound, which not only has the advantage of simplicity, but also plays an indispensable role in prostate biopsy. However, ultrasound-based radiomics studies of prostate lesions are rare. Only Wildeboer et al. reported that the multiparametric classifier combined B-mode, SWE and contrast-enhanced ultrasound (CEUS) radiomics can reach AUC of 0.75, PCa of 0.90 and great PCa, respectively (39). However, this study used unique radiomics characteristics that were inconsistent with the characteristics adopted by most studies. In our study, a more widely used Pyradiomic approach (40) was used to establish a combined model of prostate cancer diagnosis by combining radiomics with clinical factors associated with the diagnosis of prostate cancer. It can be seen that adding RAD-SCORE to the clinical model can improve the diagnostic efficiency and clinical net benefit in PCa diagnosis. As such, the model based on radiomics characteristics is obviously valuable in diagnosing PCa.

Lately, the field of clinical medicine has widely applied the nomogram figure forecast model. A lot of researches associated with this model have been published in the clinical journals with high impacts (41, 42). The nomogram figure forecast model represents a variety of disease risk elements and predicts the prognosis of patients by using risk score, which is more distinct, simple, and easy to understand. Meanwhile, after being used in clinical work effectively, it is conducive to physician–patient communication and an improved physician–patient relationship. In this study, the developed nomogram of the clinical-radiomics combined model offered an intuitive and convenient approach for physicians to diagnose PCa, and will be a new method of auxiliary diagnosis in clinical work.

### Limitations

Although the proposed method was used to improve the performance of discrimination between malignant and benign prostate lesions, several malignant ROIs were missed, and several benign ROIs were wrongly categorized as malignant. In the future, the nature of false readings (39) may be clarified further by immunohistochemical techniques. It has been suggested that prostatitis or prostatic hyperplasia, which sometimes occurs simultaneously with prostate cancer, is also considered to promote angiogenesis and change the stiffness of prostate tissue, which may attribute to the false characterization (43, 44). Future analysis including CEUS parameters may help us find more radiomics features of the multiparameter model.

Despite the hopeful outcomes, there were several limitations in this research. Firstly, our results showed that there was no statistical difference in RAD-SCORE among patients with different Gleason scores, which may be due to the small number of enrolled patients. Moreover, this study was a retrospective study conducted at a single institution. Although cross-validation was used to test the model, better evidence for clinical application needs to be obtained by multi-center validation with a larger sample size. Secondly, the Peripheral zone (PZ) and Transition zone (TZ) of the prostate were not separated in this research due to highly malignant diseases in both regions. However, there may be differences between the two regions, including in B-mode ultrasound and SWE (35). Therefore, further investigation is required to expand the size of the research object and distinguish PZ from TZ of the prostate in different ways.

## Conclusions

In our study, we developed a radiomics model to discriminate between benign and malignant lesions of the prostate with high diagnostic power and clinical net benefit. Moreover, we proved the feasibility of a multiparametric ultrasound classifier to improve the PCa localization. Using the nomogram to comprehensively consider the radiomics features and clinical factors can provide radiologists with a quantitative and intuitive method to predict PCa with more confidence. Our goal is to further expand the data set so that the performance of the model can be consolidated.

## Data Availability Statement

The raw data supporting the conclusions of this article will be made available by the authors, without undue reservation.

## Ethics Statement

The studies involving human participants were reviewed and approved by the Ethics Committee of Aerospace Center Hospital. The patients/participants provided their written informed consent to participate in this study.

## Author Contributions

LL and XZ contributed equally to this work. They jointly completed the study design, data collection, statistical analysis, and article writing. YS and HL mainly completed the data collection work. JX and JW completed the Radiomics analysis and chart making. All authors contributed to the article and approved the submitted version.

## Funding

This study is supported by Hospital level project of Aerospace Center Hospital (YN202105) and Scientific research and cultivation plan of health development in Haidian District (HP2021-32-50702).

## Conflict of Interest

Author JX was employed by Beijing Deepwise & League of PHD Technology Co., Ltd.

The remaining authors declare that the research was conducted in the absence of any commercial or financial relationships that could be construed as a potential conflict of interest.

## Appendix 1. Formulas of three radiomics models

B-mode RAD-SCORE: −0.1832+wavelet-HLH_glszm_ZoneEntropy@b×0.5937–logarithm_firstorder_Kurtosis@b×0.5746–wavelet-HLL_firstorder_Minimum@b×0.5204+wavelet-HHH_glszm_ZoneEntropy@b×0.5094–exponential_glszm_SmallAreaHighGrayLevelEmphasis@b×0.4606–wavelet-HHH_firstorder_Kurtosis@b×0.4558+wavelet-HHL_gldm_DependenceNonUniformityNormalized@b×0.4526–wavelet-LHL_gldm_SmallDependenceLowGrayLevelEmphasis@b×0.44+wavelet-LHH_glszm_GrayLevelVariance@b×0.3751–wavelet-HHH_glcm_MaximumProbability@b×0.3649–wavelet-HHL_firstorder_Mean@b×0.3207–wavelet-LHL_gldm_DependenceVariance@b×0.3118+wavelet-HHH_glszm_SmallAreaHighGrayLevelEmphasis@b×0.3083–wavelet-HHH_glszm_ZonePercentage@b×0.2929+original_shape_Elongation@b×0.2905+wavelet-HHH_glrlm_LowGrayLevelRunEmphasis@b×0.2846+wavelet-HLL_gldm_DependenceNonUniformityNormalized@b×0.2809+ gradient_glszm_SizeZoneNonUniformityNormalized@b×0.2713–wavelet-HHH_firstorder_Mean@b×0.2596+square_glszm_LowGrayLevelZoneEmphasis@b×0.2353

SWE RAD-SCORE: −0.0836–wavelet-LLH_glszm_ZoneEntropy@swe×0.8769+wavelet-HHH_glszm_ZoneEntropy@swe×0.6888+wavelet-LLL_gldm_LargeDependenceHighGrayLevelEmphasis@swe×0.5955–wavelet-HLL_glszm_LargeAreaLowGrayLevelEmphasis@swe×0.5776–gradient_firstorder_10Percentile@swe×0.4288–wavelet-HHH_firstorder_Mean@swe×0.38–wavelet-LHH_glcm_MaximumProbability@swe×0.3763–wavelet-HLL_firstorder_Skewness@swe×0.3663+wavelet-LHH_glszm_ZoneEntropy@swe×0.3531+wavelet-LHH_glrlm_GrayLevelVariance@swe×0.3201+original_glszm_SizeZoneNonUniformityNormalized@swe×0.3135–wavelet-HHH_firstorder_Skewness@swe×0.2899+wavelet-HHL_glcm_ClusterShade@swe×0.2702–logarithm_glrlm_RunLengthNonUniformity@swe×0.2654–logarithm_glszm_SmallAreaLowGrayLevelEmphasis@swe×0.2582–wavelet-HLL_glcm_MaximumProbability@swe×0.2419+wavelet-HHH_glrlm_ShortRunHighGrayLevelEmphasis@swe×0.2303+wavelet-LHH_glszm_SmallAreaHighGrayLevelEmphasis@swe×0.1917–wavelet-LHH_glszm_GrayLevelVariance@swe×0.1844–wavelet-LLH_glszm_ZoneEntropy×1.4254

Multiparametric RAD-SCORE: -0.2383–wavelet-LLH_glszm_ZoneEntropy@swe×0.7143–logarithm_firstorder_Kurtosis@b×0.6721–wavelet-HLL_firstorder_Minimum@b×0.6536+wavelet-HHL_gldm_DependenceNonUniformityNormalized@b×0.5464+wavelet-HHH_glszm_ZoneEntropy@swe×0.5366–gradient_firstorder_10Percentile@swe×0.532+wavelet-LHH_glszm_ZoneEntropy@swe×0.4812–wavelet-LHL_gldm_SmallDependenceLowGrayLevelEmphasis@b×0.4767–logarithm_glrlm_RunLengthNonUniformity@swe×0.4657–wavelet-HHL_firstorder_Mean@b×0.4663–exponential_glszm_SmallAreaHighGrayLevelEmphasis@b×0.4619+wavelet-LHH_glrlm_LowGrayLevelRunEmphasis@b×0.4544+wavelet-LHH_glszm_GrayLevelVariance@b×0.4479+exponential_glcm_Correlation@b×0.4148+original_glszm_SizeZoneNonUniformityNormalized@swe×0.4077+wavelet-HHH_glszm_GrayLevelNonUniformityNormalized@swe×0.3646–wavelet-LHH_glcm_MaximumProbability@swe×0.3372+gradient_glszm_SizeZoneNonUniformityNormalized@b×0.3029–logarithm_glszm_SmallAreaLowGrayLevelEmphasis@swe×0.2991–wavelet-HHH_glrlm_GrayLevelNonUniformityNormalized@swe×0.2899–wavelet-LLH_glszm_ZoneE-ntropy@swe×0.2865
